# Exploratory Analysis of Plasma Neurotensin as a Novel Biomarker for Early Detection of Colorectal Polyp and Cancer

**DOI:** 10.1007/s12672-019-00364-3

**Published:** 2019-05-15

**Authors:** Shengyang Qiu, Stella Nikolaou, Francesca Fiorentino, Shahnawaz Rasheed, Ara Darzi, David Cunningham, Paris Tekkis, Christos Kontovounisios

**Affiliations:** 1 Department of Surgery and Cancer, Imperial College London, Chelsea and Westminster Campus, 369 Fulham Road, London, SW10 9NH UK; 2 Department of Colorectal Surgery, Royal Marsden Hospital, London, UK; 3 Imperial Clinical Trials Unit, Imperial College London, London, UK

**Keywords:** Colorectal neoplasia, Biomarker, Early detection

## Abstract

Earlier detection of colorectal cancer (CRC) results in improved survival. Existing non-invasive biomarkers have suboptimal accuracy. Neurotensin (NTS) is involved in CRC carcinogenesis. This study evaluated the diagnostic potential of plasma NTS for colorectal polyps and cancers. Participants were selected based on national CRC referral guidelines. All subjects underwent colonoscopy. Average plasma concentrations were compared across different diagnostic groups. Predictors for detecting colorectal neoplasia were identified. Receiver operator characteristic (ROC) curve analysis assessed the diagnostic accuracy of NTS. An independent biobank was used as validation group. Of 165 participants, 46 had polyps or CRC. Significantly higher plasma NTS was found in the colonic neoplasia group (603.6 pg/ml vs. 407.2 pg/ml, *p* < 0.01). Risk factors for colonic polyps or cancers included Log_e_ (plasma NTS concentration) (OR, 2.73; 95% CI, 1.33–5.59, *p* < 0.01), log_e_ (Age) (OR, 15.49; 95% CI, 2.67–89.66, *p* < 0.01) and cigarette smoking (OR, 3.49; 95% CI, 1.31–9.26, *p* = 0.01). Plasma NTS had an optimal sensitivity of 60.4% and specificity of 71.6% for the diagnosis of colorectal polyps and cancers. Similar diagnostic accuracy was obtained in the validation group. Plasma NTS has the potential to be a non-invasive biomarker for colorectal neoplasia. It appears to be more accurate than existing blood markers and is unique in being able to detect precancerous polyps.

## Introduction

Colorectal cancer (CRC) is a worldwide health problem that ranks third in incidence and fourth in mortality with an estimated 1.2 million cases and 0.6 million deaths annually. The natural history of CRC allows prevention by early detection of precancerous lesions. Earlier diagnosis of CRC results in better survival with 1-year survival of 98% compared with 40% for stage I and stage IV disease respectively [1]. Strategies for earlier detection include screening and earlier recognition of cancer in symptomatic individuals. In the UK, the National Bowel Cancer Screening Programmes uses faecal occult blood (FOBT) test, flexible sigmoidoscopy and colonoscopy and virtual colonoscopy. Updated national referral guidelines for the recognition of cancer were introduced in 2016. A greater range of symptoms, with positive predictive value of 1%, was incorporated in to the referral criteria [2]. Both of the above rely on colonoscopy, which remains the gold standard for the detection of colorectal neoplasia. An accurate and non-invasive alternative has been sought.

The last decades have been marked by the accumulation of knowledge about the inner workings of the normal and abnormal cells of the colon. The discovery and validation of novel assays is the source of new diagnostic modalities. In this context, we assess the reliability of the neurotensinergic system to cancer progression as well as the regulation and mechanism of the system in order to determine its potential in colorectal cancer diagnosis, surveillance and screening.

Neurotensin (NTS) is a 13-amino-acid peptide originally isolated by Garraway and Leeman [3]. NT has shown to exert numerous oncogenic effects involved in tumour growth and metastatic spread. These effects are mostly mediated by neurotensin receptor 1 (NTSR1) [4].

NTSR1 appears to be solely expressed in colonic cancer cells but not in normal colon cells [5, 6]. The NTS/NTSR1 complex is an actor in cancer progression in human colonic adenomas and cancers [7, 8]. In in vitro studies, the addition of NT to human colon cancer cell lines resulted in significantly increased cancer cell growth and the growth of xenografted human colon cancer cells in mice [9]. In vivo, NTSR1 mRNA expression was undetectable in superficial differentiated epithelial cells in histological specimens of normal human colonic epithelium, but there was moderate and strong expression in adenomas and adenocarcinomas respectively. Tumours that infiltrated into and beyond the muscularis propria showed even higher expression [10].

Small-scale studies have shown elevated circulating NTS in peripheral blood in patients with CRC [11, 12]. A pilot study included 26 patients who underwent colonoscopy of whom 14 had colon pathology. Pathology in colon was associated with 3.7-fold increase in NTS levels. In multivariate analysis, patients with pathology in the colon have increased plasma NT levels compared with controls adjusted for age, gender, BMI and co-morbidities. After ROC curve analysis, NTS in plasma was associated with 87.5% sensitivity and 91.7% specificity for discriminating the colon pathology from normal colonic epithelium. (AUC = 0.893 95% CI; 0.749–1, *p* = 0.001). This is in comparison to CEA, the most widely used CRC biomarker, which has a sensitivity of 36% and a specificity of 87% for Dukes A and B colorectal cancers. [13] Furthermore, there are currently no blood markers for colonic polyps.

The aim of this study is to investigate whether plasma NTS could be a non-invasive diagnostic tool in the early detection of colorectal neoplasia. If validated, it could lead to the discovery of a non-invasive blood-based marker that could detect early stage. We conducted a large-scale prospective study to further investigate the diagnostic potential of plasma neurotensin as a diagnostic marker for colorectal polyps and cancers in patients who have symptoms which fulfil UK national criteria for suspected colorectal cancer [2].

## Patients and Methods

A prospective, multicentre, double-blinded cohort study was setup with ethical approval from the Human Research Authority (REC reference 16/SC/0523). Eligible participants were those referred to undergo colonoscopy for symptoms suspicious for colorectal malignancy based on national referral guidelines for suspected colorectal cancers [2] or those having colonoscopy to confirm diagnosis of colorectal cancer. Exclusion criteria were patients aged 17 years or younger, medical or psychiatric conditions impairing ability to give informed consents; patients currently receiving cancer treatment e.g. on chemotherapy; patients who were pregnant, lactating, or undergoing fertility treatment; and patients who were being actively treated for another malignancy.

All patients who were referred to the study centres were screened for eligibility. After recruitment, baseline patient demographics collected included age, sex, BMI, comorbidities, regular medication, smoking and alcohol intake and medication history. Peripheral venous blood samples were prospectively collected and were anonymised to avoid bias during sample analysis. Eight millilitres of blood was collected from each patient into EDTA bottles. These samples were collected prior to patients undergoing colonoscopy or surgery. Patients were fasting for 24 h prior to sample collection. Blood samples were centrifuged for 15 min at 1000×*g* at 2–8 °C within 30 min of collection. Plasma was immediately aliquoted and stored at − 80 °C.

Furthermore, plasma samples stored in the Imperial College Healthcare Tissue Bank (ICHTB, Human Tissue Authority Licence: 12275, Research Ethics Committee Approval: 12/WA/0196, Tissue Bank Application number R16007) were used to cross validate the findings from the prospective study. The stored samples were collected from patients who match the inclusion criteria of this study. The samples were collected and stored between 2013 and 2015. These samples were collected from non-fasting individuals.

A competitive enzyme immunoassay (ELISA) kit (catalogue number ABX152510, Abbexa Ltd., Cambridge, UK) was used to detect NTS as per the manufacturer’s instructions. Standard protocol for ELISA was followed to ensure the accuracy of the results. Standard concentration curves were constructed for each individual assay. Close fit of the curve (*R*^2^ > 0.98) was achieved for all standard curves. Each sample was assayed multiple times to ensure consistency of results.

### Outcome Measures

The primary outcome measure was the sensitivity and specificity of plasma NTS as a diagnostic marker for colorectal polyps or cancer. Secondary outcome measures were the differences in plasma NTS concentration between individuals with normal colons and different colonic pathologies, as well as risk factors for the presence of colonic pathologies in the study cohort.

### Statistical Analysis

Based on published pilot data, in order to observe a two-sided 95% confidence interval of 75–95% for a sensitivity of 85%, we needed to recruit 49 patients with colonic polyps or cancer [11]. Given reported polyp and cancer detection rates of 30%, we estimated that 164 participants needed to be recruited to the study. Assuming a loss to follow-up of 5%, we recruited 170 participants in total.

To minimise bias, researchers were blinded to the colonoscopy findings for each subject. The colonoscopic finding for each participant was retrieved from the electronic records of colonoscopy and histology reports after all sample analysis was complete. The diagnoses were categorised as abnormal, i.e. cancer, polyps (adenomatous and hyperplastic) or other benign pathology (colitis, diverticular disease), and normal.

A pre-specified statistical analysis plan was signed off before unblinding the data. Statistical analysis was performed using SPSS Statistics (version 20.0, IBM Corporation, USA). Median plasma neurotensin concentrations were compared using Mann-Whitney *U* and Kruskal-Wallis tests across diagnoses groups. Univariate binary logistic regression was performed to identify risk factors for colonic pathology on colonoscopy. Normality was checked and where variables were non-normal, a log transformation was carried out prior to regression analysis. Risk factors with significance of *p* < 0.10 were included in a multivariate regression model to identify those associated with colonic polyps/cancer. Finally, receiver operating characteristic (ROC) curve was constructed to determine the diagnostic accuracy of plasma NTS. The optimal cut-off of plasma NTS as well as the sensitivity and specificity of the test was calculated using the Youden index.

## Results

**Fig. 1:**
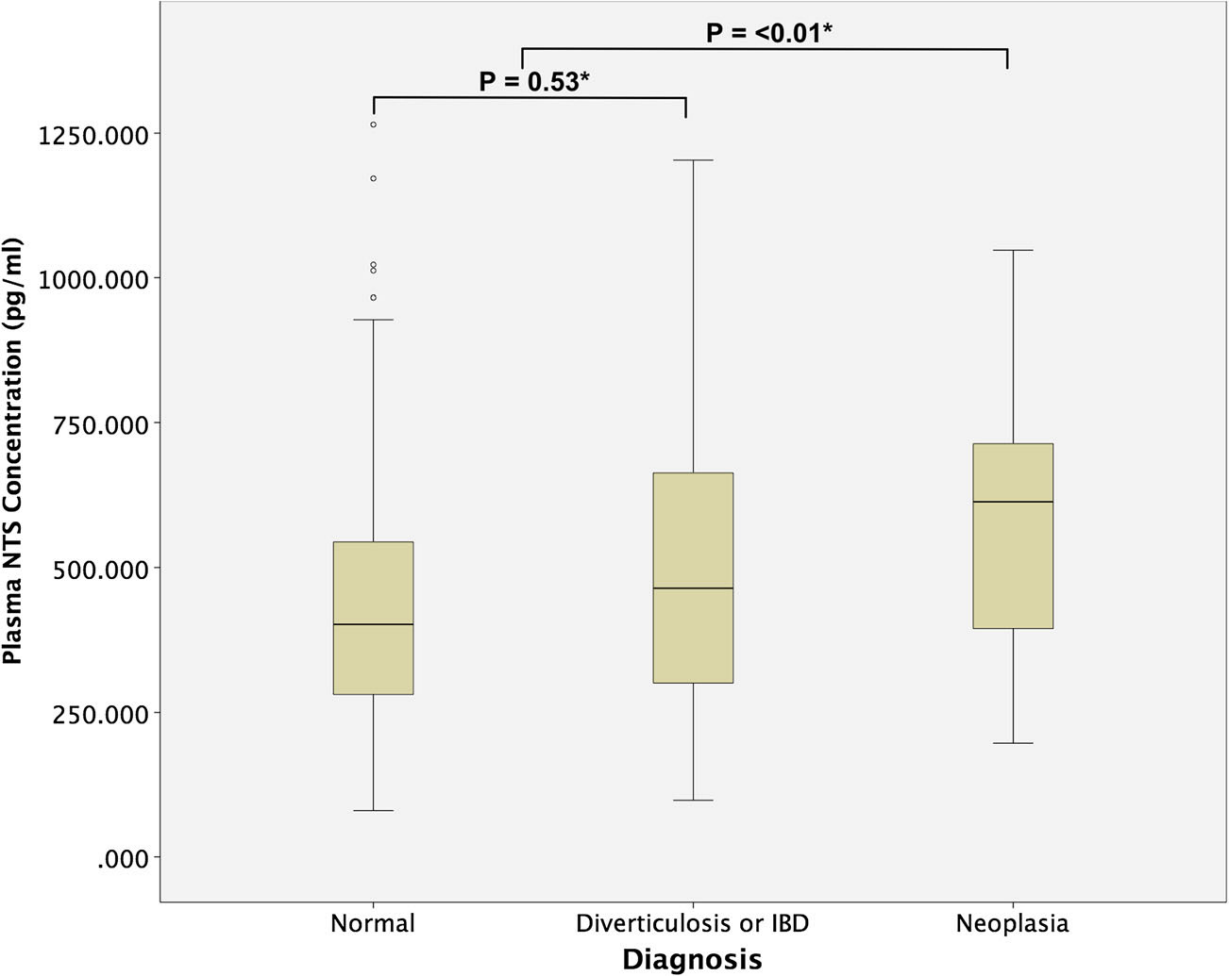
Plasma neurotensin concentration across different colorectal pathologies on colonoscopy

One hundred and eighty patients were recruited between November 2016 and July 2017. Fifteen did not complete colonoscopy or were unable to provide plasma sample for analysis. All blood samples provided were taken immediately prior to undergoing colonoscopy. The median age of the remaining 165 patients was 61.5 years with an interquartile range (IQR) of 51.8–61.8 years. Eighty-four patients had normal colonoscopies and 81 had colonic pathologies. Of these, 46 had either colonic polyps or cancers. The median plasma NTS concentration and patient demographics between diagnostic groups are shown in Table 1 and Fig. 1. There were no adverse events from performing the blood test or reference tests. There was a significant difference between the plasma NTS concentrations across the diagnoses groups (*p* = 0.04, Kruskal-Wallis test). There was a significant difference between NTS concentration in individuals with normal colons (median 401.6 pg/ml) and those with colonic pathologies (median 550 pg/ml). (*p* = 0.03, Mann-Whitney *U* test). However, there was no significant difference between the normal colonoscopy group and the group of patients found to have colonic diverticulosis or inflammation. Therefore, the difference observed between the normal and abnormal colonoscopy groups was a result of the difference in NTS concentration of individuals with colonic neoplasia (polyps or cancers) and those without (Table 2).

**Table 1:** Patient demographics and median plasma neurotensin concentration across diagnosis groups. *IQR* inter quartile range, *ASA* American Society of Anesthesiology grade, *pg/ml* picograms per millilitre

Diagnosis	Number of cases	Median age (IQR)	Sex (male:female)	Median ASA	Smoker (%)	Median BMI	Median plasma neurotensin concentration (pg/ml) (IQR)
Normal	84	55.1 (50.6–66.8)	37:47	2	12.9	24.5	402.1 (280.7–601.8)
Diverticulosis	19	67.0 (60.6–72.6)	7:12	2	11.8	23.3	464.1 (300.1–714.0)
Colonic inflammation	8	56.6 (42.4–65.2)	4:4	1	14.3	29.0	408.6 (234.2–675.7)
Hyperplastic poly (s)	7	60.1 (54.2–72.3)	4:3	1	40.0	27.1	536.3 (340.5–633.3)
Adenomatous poly (s)	26	67.4 (61.2–78.2)	8:18	2	44.4	23.5	641.2 (507.6–774.7)
Cancer	20	64.3 (46.9–72.1)	11:9	2	18.8	24.5	530.3 (283.6–712.5)

**Table 2:** Median plasma concentration between diagnostic groups. *Independent Mann-Whitney *U* test. No colonic neoplasia group includes normal, diverticulosis and colonic inflammation. Colonic neoplasia group includes hyperplastic polyps, adenomatous polyps and colorectal cancer

Diagnosis	Median plasma neurotensin concentration (pg/ml) (IQR)	*p*
Normal	401.6 (267.6–654.4)	0.03*
Abnormal	550.6 (339.6–712.5)	
Normal	401.6 (267.6–654.4)	0.53*
Diverticulosis or IBD	464.1 (299.9–714.0)	
No colonic neoplasia	407.2 (287.3–648.5)	< 0.01*
Colonic neoplasia	603.6 (394.0–714.8)	

The median plasma NTS level in those with colonic polyps (hyperplastic or adenomatous) or cancer was 603.6 pg/ml, which was significantly different to individuals without polyps or cancer (407.2 pg/ml) (*p* < 0.01, independent samples Mann-Whitney *U* test). After natural log transformation, significant difference remained between the polyp or cancer group and the non-polyp or cancer group (*p* < 0.01, independent samples Student *t* test).

There was no significant difference between the NTS plasma concentration in patients with polyps or cancer. (*p* = 0.215, Mann-Whitney *U* test). When the NTS plasma concentration was compared between patients with colonic polyps or cancer in different locations within the colon (right, transvers, left, and rectum), there was no significant difference (*p* = 0.09, Kruskal-Wallis Test).

Univariate binary logistic regression analysis showed age, smoking status and elevated plasma NTS were significant risk factors for detecting colorectal polyps or cancers on colonoscopy (Table 3). Multivariate binary regression analysis that demonstrated Log(Plasma NTS) was an independent risk factor for detecting colorectal neoplasia on colonoscopy (*p* < 0.01, OR 2.73, 95% CI 1.33–5.59) (Table 4).

**Table 3:** Univariate binary logistic regression for risk factors for colonic polyps or cancers. *Binary logistic regression analysis. ^**+**^Natural logarithmic transformation for non-normal variables applied. *β* beta coefficient, *S.E*. standard error, *95% CI* 95% confidence interval

	β	S.E.	Odds ratio	95% CI	*p* value
Sex (male)	0.00	0.34	1.00	0.51–1.05	1.00*
*Log*_*e*_ (*age*)	*1.94*	*0.80*	*6.95*	*1.45–33.30*	0.02* ^*+*^
BMI	0.00	0.03	1.00	0.94–1.07	0.93*
ASA	0.15	0.19	1.16	0.76–1.70	0.44*
*Smoker*	*0.96*	*0.44*	*2.61*	*1.01–6.23*	0.03*
Log_e_ (units per week of alcohol)	0.52	0.31	1.68	0.91–3.08	0.10*^+^
*Log*_*e*_ (*plasma neurotensin concentration*)	*0.90*	*0.35*	*2.47*	*1.24–4.85*	0.01* ^*+*^

*p* values are significant < 0.05

**Table 4:** Multivariate regression of risk factors for colonic polyps or cancers. *β* beta coefficient, *S.E.*, standard error, *95% CI* 95% confidence interval

	β	S.E.	Odds ratio	95% CI	*p* value
Log_e_ (age)	2.74	0.90	15.49	2.67–89.66	< 0.01
Log_e_ (plasma neurotensin concentration)	1.004	0.37	2.73	1.33–5.59	< 0.01
Smoker	1.249	0.499	3.49	1.31–9.26	0.01

**Fig. 2:**
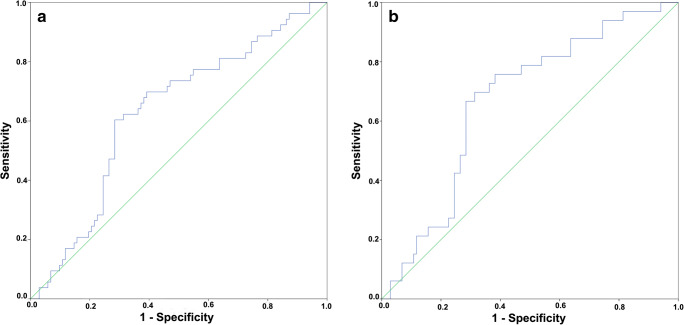
Receiver operator characteristic curves for diagnostic accuracy of plasma neurotensin for detecting colorectal polyps and cancers (**a**) and colorectal polyps (**b**)

ROC curve analysis of the diagnostic accuracy of plasma NTS for detecting colonic polyps or cancers showed an area under the curve (AUC) of 0.63 (*p* < 0.01) (Fig. 2a). The optimal cut-off was 550 pg/ml with a sensitivity of 60.4% and specificity of 71.6%. The AUC for detecting colonic polyps was 0.67 (*p* < 0.01) with optimal cut-off of 534 pg/ml. The sensitivity was 69.7% and specificity of 68.6% (Fig. 2b).

Analysis of 100 plasma samples obtained from the tissue bank again revealed significant difference between the plasma concentration of NTS between diagnostic groups (*p* = 0.03, Kruskal-Wallis test). The median NTS concentration of patients with colonic neoplasia was 457 pg/ml compared with 338.9 pg/ml in those without polyps or cancer (*p* = 0.03, Mann-Whitney *U* test).

The median concentration of plasma NTS was 368.0 pg/ml in the tissue-banked samples compared with 447.6 pg/ml in the prospectively collected sample (*p* < 0.01, Mann-Whitney *U* test), similar diagnostic accuracy of plasma NTS for detecting colorectal polyps and cancers with the prospective cohort. The AUC was 0.69 (*p* < 0.01) with optimal sensitivity of 66.7% and specificity of 62.9% albeit with a lower cut-off of 376 pg/ml compared with the prospective cohort (Appendix Table 5).

## Discussion

Currently, it has been estimated that there were 136,830 new cases of colorectal cancer (CRC) and 50,310 deaths due to CRC in the USA in 2014. In the European Union (EU), colorectal cancer is the third most common cancer and the second leading cause of death, accounting for more than 345,000 new cases and 150,000 deaths in 2012. Survival is highly dependent on early diagnosis. Early detection of CRC can improve the 5-year survival rate from about 10% in stage IV disease to up to 90% in stage I disease [14]. Additionally, the adenoma to adenocarcinoma and serrated pathways in CRC is linear. Early detection of non-cancerous colonic lesions with prompt removal followed by surveillance can significantly reduce the incidence of CRC.

Colonoscopy remains the gold standard for diagnosis and screening of colorectal polyps. However, its invasive nature carries with it complications such as colonic perforation and bleeding. It also carries with it significant economic costs [15]. However, there appears to be high non-compliance rates in countries with colonoscopic bowel screening programmes [15, 16]. FOBT and FIT are cheaper and non-invasive alternatives for screening of colorectal cancer. These have 60 to 80% sensitivity and 85 to 95% specificity [17]. For adenomas, the sensitivity and specificity for FIT was 18% and 95% [18]. Adherence to repeated FOBT is paramount in ensuring the robustness of screening but adherence to repeated FOBT is low [19]. There also appears to be a bias for poorer participation in lower socieoeconomic groups, when it is known that those in lower socioeconomic groups who present with CRC have more advanced disease and worse outcomes [20, 21]. Blood markers such as CEA and cancer antigen (CA) 19–9 have sub-optimal sensitivity and specificity, are exclusively used as surveillance markers and are excluded from the screening recommendations [22]. More promising results have been published for genes deriving from peripheral blood mononuclear cells (PBMCs) including DNA methylation of specific genes, but validation in the clinical settings is awaited [23, 24].

The ideal marker should have the following qualities: high sensitivity and specificity, it should be safe and non-invasive so that it can be broadly accepted by patients, cost effectiveness so that it can be easily adopted, easy to measure and should be detected among different genders and ethnic groups [25–27].

Some candidate proteins have been published as CRC diagnostic markers. A single protein marker, TIMP-1, has detected CRC with 42–65% sensitivity and 95% specificity [28]. Babel et al. reported 43 proteins that could distinguish between CRC patients and healthy controls [29]. These are awaiting clinical validation.

NTS and its receptors have been implicated in the progression of a broad range of human cancers. These include cancers of the breast, prostate, lung, liver and pancreas among others [30–34]. There is compelling evidence for the involvement of NTS and its receptors in the oncogenesis of human colorectal cancer [35]. NTS appears to promote cell growth in CHO cells transformed with human NTSR1 and in colon cancer HT29 cells [36]. It also stimulates growth in five different human cancer lines (SW480, SW620, HT29, HCT116 and C1.19A) that express NTSR1, but has no effect on cells with absent NTSR1 [5]. Gui et al. examined NTSR1 mRNA by in situ hybridization in normal colonic mucosa, adenomas and colonic adenocarcinomas. NTSR1 mRNA expression was found to be non-detectable in epithelial cells of normal colonic epithelium but adenomas and adenocarcinomas demonstrated moderate to strong expression (*p* < 0.05). There was higher level of expression in adenocarcinomas compared with adenomas (*p* < 0.05) [10].

This study is the first large-scale prospective clinical validation of plasma NTS level for the diagnosis of colonic polyps and cancers. The study demonstrated significantly higher plasma NTS levels in individuals with colonic neoplasia. NTS appears to have a sensitivity and specificity of 60 to 70% for colorectal cancers and polyps compared with CEA, where only 4% of patient with stage I CRC had elevated plasma levels [37]. It also appears to be unique in its ability to differentiate patients with colonic polyps with those without polyps.

Plasma NTS appears to have some potential as a screening, diagnostic and surveillance marker for colonic polyps and cancers. It has advantages over existing tests such as FBOT in its ease of use (single blood test versus multiple stool samples), as well as the ability of detecting pre-cancerous lesions in the colon. The result of this study also suggests the diagnostic ability is not affected by the fasting status of patients’ plasma concentration from the biobanked samples, from patients who were not fasted, and had similar diagnostic efficacy compared with patients who were fasted in the prospectively collected sample. This study is also feasible to perform the assay in plasma samples that have been stored. Having said that, there is a clear difference between the plasma NTS concentration and these patient cohorts. This could be due to the aforementioned difference in fasting status or possibly due to the degradation of plasma NTS in stored samples. Further work is clearly needed to investigate the effect of fasting and storage on plasma NTS concentrations.

In both cohorts of samples, there was significant variation in levels of plasma NTS between individuals. There appears to be subsets of individuals with colonic polyps or cancers who do not have high levels of plasma NTS and individuals with high plasma NTS without any colonic lesions. This ultimately limits the diagnostic accuracy of plasma NTS. Further work is needed to clearly understand relationship between NTS expression in colonic neoplasia and its plasma concentration. In the multivariate regression model, NTS concentration was an independent risk factor for colonic polyps and cancers, along with cigarette smoking and advancing age. There may be other confounding factors which may result in differential expression of plasma NTS in individuals, understanding the cause versus effect relationship between presence of colonic neoplasia, and plasma NTS is important in improving the diagnostic ability of NTS. For example, does a high level of baseline NTS expression predisposes a certain individual to colonic hyperplasia and metaplasia, or are colonic neoplasia responsible for directly secreting or stimulating the secretion of NTS into blood. Multi-centre studies powered to include larger number CRC and polyps with concurrent examination of genetic expression of relevant genotypes as well as histological examination of expression of NTS and NTS receptors on neoplastic tissue are required to answer these questions.

As a result of the small number of CRC patients recruited into the study, it was not possible to determine if there are any significant differences of plasma NTS concentration in individuals with CRC and those with polyps. In the prospective cohort, the NTS concentration appeared lower in the CRC group compared with the polyp group. This is in contrast to the findings in the biobanked samples. The inclusion criteria for this study were all patients who were referred for suspected CRC based on national guidelines. The low positive predictive values of the referral criteria resulted in low CRC diagnostic yield. The CRC cases were also heterogeneous in terms of their location in the colon as well as histological grade. Both of these factors may affect plasma NTS level. Continued sample collection in order to obtain an adequate sample of CRC is required to draw conclusions.

In order to evaluate the potential of using plasma NTS as a screening marker, future studies must evaluate this marker in the setting of asymptomatic individuals. Likewise, its use as a surveillance marker will require serial evaluation of plasma NTS in individuals before and after treatment of colorectal neoplasia (i.e. polypectomy or CRC resection).

## Conclusion

NTS plasma concentration appears to have the potential to differentiate individuals with colonic neoplasia with those who do not. Furthermore, it has the potential to detect patients with colorectal polyps as well as CRC. The use of NTS blood test as a diagnostic adjunct for colonic polyps and cancers could offer an advantage over existing diagnostic modalities. Therefore, it warrants further validation, along the biomarker development roadmap, in order for it to be incorporated into future clinical practice.

## Appendix

**Table 5:** Plasma neurotensin concentrations across diagnostic groups of biobanked plasma samples

Diagnosis	Number of cases	Median plasma neurotensin concentration (pg/ml) (IQR)
Normal	46	338.9 (214.9–467.6)
Diverticulosis	9	321.3 (203.6–495.2)
Colonic inflammation	7	345.0 (214.7–433.7)
Adenomatous poly (s)	25	439.6 (313.8–629.2)
Cancer	8	549.3 (344.5–1152.0)
